# Dataset of the physical conditions of Green Ash (*Fraxinus pennsylvanica*) in riparian woodlands along the central Platte River

**DOI:** 10.1016/j.dib.2018.10.063

**Published:** 2018-10-24

**Authors:** Joshua D. Wiese, Andrew J. Caven

**Affiliations:** The Crane Trust, 6611 W Whooping Crane Dr., 68883 Wood River, NE, USA

**Keywords:** Green Ash, Emerald Ash Borer, Riparian woodlands, Platte River

## Abstract

The 2016 discovery of Emerald Ash Borer (*Agrilus planipennis; EAB*) in Nebraska warranted an assessment of the physical conditions of ash trees (*Fraxinus spp.*) across the state. Here we present a dataset of current physical conditions and spatial location of 30 Green Ash (*Fraxinus pennsylvanica*) throughout riparian woodlands along the Platte River in southcentral Nebraska. Ten Green Ash were assessed along transect lines through three riparian woodlands. Physical indicators of EAB infection and morphometric characteristics were recorded at each tree including diameter at breast height (DBH), estimated age, canopy condition rating scale (CCRS), borer holes, woodpecker holes, serpentine gallery, epicormic shoots, basal shoots, and bark splitting to document the health and condition of local Green Ash. We recorded variables of crown class category (CCC), crown ratio, and Green Ash seedling and sapling numbers within 6 m of the measured tree to document current canopy cover and regenerative potential of Green Ash within each woodland. Metric summaries are provided looking at each woodland individually and at the woodlands combined.

**Specifications table**

**Table:** 

Subject area	*Biology*
More specific subject area	*Riparian forestry*
Type of data	*Microsoft Excel File (Comma Separated Values, CSV file)*
How data was acquired	*Field survey*
Data format	*Raw*
Experimental factors	*Field data: the diameter and physical conditions of Green Ash trees*
Experimental features	*In three separate woodlands, 30 individual Green Ash were measured for diameter at breast height (dbh), forest stand data, and physical stress associated with insect infestation was recorded*
Data source location	*Crane Trust, Hall County, Nebraska, USA*
	*N 40.79763°, W -98.45416°N 40.79529°, W -98.39854°N 40.80163°, W -98.43842°*
Data accessibility	*With this article*
Related research article	*Burr, S.J., and D.G. McCullough. 2014. Condition of green ash (Fraxinus pennsylvanica) overstory and regeneration at three stages of the emerald ash borer invasion wave. Canadian Journal of Forest Research 44(7): 768–776.*

**Value of the data**

• This dataset provides the location, size, estimated age, and physical condition of Green Ash in the region with which future data can be compared.
• Crown Class Category data describes the relative dominance and ecological role of Green Ash trees within three riparian woodlands at a distinct time period.
• The dataset documents physical symptoms caused by EAB and other insects, many of which have been standardized in forestry and EAB monitoring protocols, and can be used as a comparison in other tree health studies.
• The current absence of EAB is documented, which can be used as a reference for changing distributions.
• This dataset represents a sample of Green Ash characteristics in the region prior to EAB invasion and can serve as a record for future restoration efforts concerning the species.

## 1 Data

**Fig. 1:**
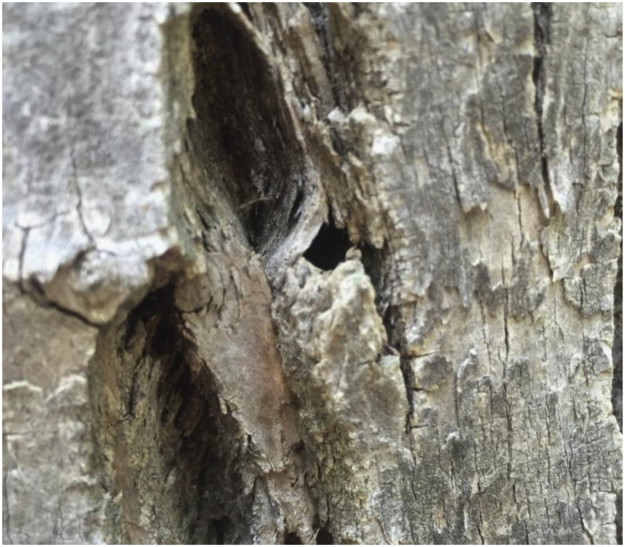
Borer hole found on Green Ash identified as a native borer species by UNL entomologist.

**Fig. 2:**
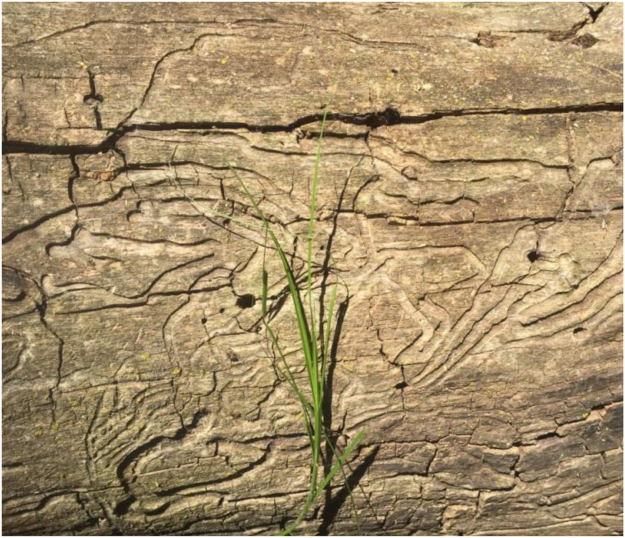
Serpentine gallery found on Green Ash identified as native borer species by UNL entomologist.

The dataset (WieseCaven_GreenAshHealth_CPRV2016.csv; Metadata in Table 1) provides current locations and inventory of the physical condition of 30 living Green Ash trees in the central Platte River Valley from 2016. The dataset was sampled from three riparian woodlands totaling 15.5 ha in size (10 trees per woodland). We strategically sampled Green Ash along three 100–125 m length transects to best represent the diversity of age classes present within each woodland habitat. We recorded physical symptoms related to potential EAB damage [1] and other metrics related to the Green Ash dominance in each woodland. Borer hole (*n* = 2/30 trees; Fig. 1) and serpentine gallery (*n* = 2/30 trees; Fig. 2) damage was determined not to be caused by EAB by a University of Nebraska-Lincoln entomologist. An explanation of all data variables is provided (Table 1) and a descriptive summary of each variable is provided for each woodland and the woodlands combined (Table 2). Data demonstrates a variable age among woodlands with Calving Pasture Woodland (CPW) and Big Slough Woodland (BSW) averaging 18 years older and about 13 cm wider in diameter at breast height (DBH) than the Southeast Mormon Woodland (SEMW) (Table 2). Accordingly, a higher percent of trees in CPW (50%) and BSW (40%) were categorized as dominant within the forest canopy compared to SEMW (20%).

**Table 1:** Metadata explaining variable codes for the raw dataset (Excel Table 1) and for the descriptive summary dataset (Table 2).

*Variable Code*	*Description*	*Variable Type*
*LotID*	*Assigned woodland code, followed by a number that corresponds to the metal tag number placed on each tree.*	*Nominal*
	*e.g. Big Slough Woodland tree tag number 60 = BSW60*	
*Latitude*	*Latitude of the documented tree*	*Continuous*
*Longitude*	*Longitude of the documented tree*	*Continuous*
*DBH*	*Diameter in centimeters at breast height of each documented tree*	*Continuous*
*Age*	*Calculated Green Ash age estimation**[5]**:*	*Continuous*
	*Age (years) = DBH (inches) × 3.5*	
*CCRS*	*Ash Canopy Condition Rating Scale*[1], [2]:	*Ordinal*
	1. *“Canopy is full and healthy”* 2. *“Canopy has started to lose leaves (thinning), but no dieback (dead top canopy twigs without leaves) is present”* 3. *“Canopy has less than 50% dieback”* 4. *“Canopy has more than 50% dieback”* 5. *“Canopy has no leaves, epicormic sprouts may be present on the trunk”*	
*Borer Holes*	*Number of "D" shaped holes caused by borers found between 1.25 and 2.25 m on tree trunk*	*Count*
*WP Holes*	*Number of larger woodpecker holes found between 1.25 and 2.25 m on tree trunk*	*Count*
*Gallery*	*Presence of "S" shaped serpentine galleries beneath peeled bark, caused by borer larvae*	*Nominal*
*Epic Shoots*	*Presence of new foliate branches on the tree trunk* (*Epicormic shoots), signifying tree stress*	*Nominal*
*Basal Shoots*	*Presence of new foliate branches at the base of the tree (basal shoots), signifying tree stress*	*Nominal*
*Bark Split*	*Vertical separation of bark from trunk and exposing cambium*	*Nominal*
*CCC*	*Crown class category for each tree representing the relative dominance of Green Ash*[1], [4]*:*	*Categorical*
	*D – “Dominant- Above general canopy of stand, receives direct sunlight on top and all sides”*	
	*C – “Codominant- Average position in stand, receives direct sunlight on top and at least one side”*	
	*I – “Intermediate- Below general canopy, receives direct sunlight on top”*	
	*S – “Suppressed- Completely overtopped; receives no direct sunlight”*	
*Crown Ratio*	*Proportion of the tree׳s living crown in comparison to the total height of the tree*	*Continuous*
*Seedling #*	*Number of Green Ash <1 m tall within a 6 m radius of the documented tree*	*Count*
*Sapling #*	*Number of Green Ash 1–2 m tall within a 6 m radius of the documented tree*	*Count*

**Table 2:** Descriptive summaries of measured variables for individual woodlands and all woodlands combined. Count data for Seedling # and Sapling # were combined (Seedling + Sapling) for summaries. Variable average, standard deviation (SD), and the minimum and maximum (min-max) of continuous ordinal and count data are given. Nominal variables were summarized by percentage (%). Categorical variables were summarized by percentage of trees in each category (e.g. Percent dominant Crown Class Category (CCC) = “D” CCC %).

LotID	CPW	SEMW	BSW	All Woodlots
*DBH Average*	42.3	29.1	42.1	37.8
*DBH min-max*	29.3–58.9	5.7–42.7	23.9–63.7	5.7–63.7
*DBH SD*	9.0	12.1	15.4	13.6
*Age Average*	58	40	58	52
*Age min-max*	40–81	8–59	33–88	8–88
*Age SD*	12.5	16.6	21.3	18.7
*CCRS Average*	2.2	1.8	2.3	2.1
*CCRS min-max*	1–4	1–3	1–4	1–4
*CCRS SD*	1.0	0.9	0.9	1.0
*Gallery %*	10%	0%	10%	7%
*Epic Shoots %*	30%	60%	10%	33%
*Basal Shoots %*	20%	10%	10%	13%
*Bark Split %*	10%	10%	10%	10%
*"D" CCC %*	50%	20%	40%	37%
*"C" CCC %*	40%	60%	40%	47%
*"I" CCC %*	10%	10%	20%	13%
*"S" CCC %*	0%	10%	0%	3%
*Crown Ratio Average*	0.54	0.73	0.62	0.63
*Crown Ratio min-max*	0.11–0.87	0.53–0.89	0.43–0.82	0.11–0.89
*Crown Ratio SD*	0.21	0.13	0.13	0.17
*Seedling + Sapling Average*	0.6	0.4	0.0	0.3
*Seedling + Sapling min-max*	0.0–2.0	0.0–4.0	0.0–0.0	0.0–4.0
*Seedling + Sapling SD*	0.7	1.3	0.0	0.8

## 2 Experimental design, materials, and methods

The survey area consisted of three riparian woodlands along the Platte River that are owned and managed by the Crane Trust, Hall County, NE, USA (www.cranetrust.org). Woodlands, namely Big Slough Woodland (BSW), Calving Pasture Woodland (CPW), and Southeast Mormon Woodland (SEMW), contained established Green Ash trees to be sampled. Transect lines were strategically placed in areas of the woodlands where Green Ash was known to be present and continued 100–125 m at a consistent bearing from woodland edges moving toward their interiors. Transect bearings and distances for woodland were as follows: 125 m at 252 degrees (magnetic bearing) for SEMW, 110 m at 110 degrees for CPW, and 105 m at 180 degrees for BSW. Ten Green Ash trees > 2 m tall were sampled within 15 m of either side of the transect line in each woodland. However, trees outside of this area were sampled when necessary to best represent each woodlands’ demographic composition. We attempted to find a representative sample of age classes along each transect. The GPS coordinates of each Green Ash surveyed were recorded and marked with a numbered metal tag providing the means to monitor individual trees over time. This dataset serves as a record of the current status of Green Ash in the area in the likely event of species decline from EAB.

Health metrics associated with EAB [1] and other insect damage on Green Ash were taken for each tree (Excel Table 1). Diameter at breast height (DBH), canopy condition rating scale (CCRS) [1], [2], borer holes, woodpecker holes, serpentine gallery, epicormic shoots, basal shoots, and bark splitting were recorded to document an individual Green Ash׳s health and condition [1]. Of the Green Ash trees surveyed (*n* = 30) only 2 showed signs of borer holes, though these holes were determined to be native ash borer holes and not Emerald Ash Borer [3]. Forest stand data [4] was also taken regarding current canopy composition and condition of each Green Ash tree (Excel Table 1). Crown class category (CCC), crown ratio, estimated age from DBH [5], and Green Ash seedling and sapling number within 6 m of the measured tree was assessed as a record of current canopy cover and regenerative potential of Green Ash. We also provide descriptive metric summaries of all woodlands individually and combined to represent the current state of Green Ash in the region (Table 2).
