# Supplementary material for: Dataset of the physical conditions of Green Ash (Fraxinus pennsylvanica) in riparian woodlands along the central Platte River
Source: Data Brief. 2018 Oct 24;21:948–52. doi: 10.1016/j.dib.2018.10.063 (PMC6222171; doi:10.1016/j.dib.2018.10.063)
Supplement: Supplementary file 1 — Transparency document [file mmc1.pdf]

DISCLOSURE OF POTENTIAL CONFLICT-OF-INTEREST

All accepted articles will be published only after the signed disclosure statements have been completed. The information will be published as a footnote to the article on the Title page.

Please respond to each of the following questions by checking the appropriate boxes.

Each author must complete his/her own form.

1. Did the author of the manuscript receive funding, grants, or in-kind support in support of the research or the preparation of the manuscript?

[x] NO

[ ] YES, support received from the following persons, agencies, industrial or commercial parties is disclosed below.

If yes, did the support include contractual or implied restriction on utilization or publication of the data and/or review of the data prior to publication?

[ ] NO

[ ] YES

2 Did the author have association or financial involvement (i.e. consultancies/advisory board, stock ownerships/options, equity interest, patents received or pending, royalties/honorary) with any organization or commercial entity having a financial interest in or financial conflict with the subject matter or research presented in the manuscript?

[x] NO

[ ] YES, the association or financial involvement of the authors is disclosed below.

1.
2.
3.

(Use additional sheets if necessary)

Joshua Wiese

Author Name/Signature:

Title of Article: Dataset of physical conditions of Green Ash (Fraxinus pennsylvanica) in riparian woodlands along the central Platte River

Manuscript Number: DIB-D-18-02265

Date: October 11, 2018

DISCLOSURE OF POTENTIAL CONFLICT-OF-INTEREST

All accepted articles will be published only after the signed disclosure statements have been completed. The information will be published as a footnote to the article on the Title page.

Please respond to each of the following questions by checking the appropriate boxes.

Each author must complete his/her own form.

1. Did the author of the manuscript receive funding, grants, or in-kind support in support of the research or the preparation of the manuscript?

[x] NO

[ ] YES, support received from the following persons, agencies, industrial or commercial parties is disclosed below.

If yes, did the support include contractual or implied restriction on utilization or publication of the data and/or review of the data prior to publication?

[ ] NO

[ ] YES

2 Did the author have association or financial involvement (i.e. consultancies/advisory board, stock ownerships/options, equity interest, patents received or pending, royalties/honorary) with any organization or commercial entity having a financial interest in or financial conflict with the subject matter or research presented in the manuscript?

[x] NO

[ ] YES, the association or financial involvement of the authors is disclosed below.

1.
2.
3.

(Use additional sheets if necessary)

Andrew Caven

Author Name/Signature:

Title of Article: Dataset of physical conditions of Green Ash (Fraxinus pennsylvanica) in riparian woodlands along the central Platte River

Manuscript Number: DIB-D-18-02265

Date: October 11, 2018
